# Different Pathways Mediate Amphotericin-Lactoferrin Drug Synergy in *Cryptococcus* and *Saccharomyces*

**DOI:** 10.3389/fmicb.2019.02195

**Published:** 2019-10-01

**Authors:** Yu-Wen Lai, Chi Nam Ignatius Pang, Leona T. Campbell, Sharon C. A. Chen, Marc R. Wilkins, Dee A. Carter

**Affiliations:** ^1^School of Life and Environmental Sciences, The University of Sydney, Sydney, NSW, Australia; ^2^School of Biotechnology and Biomolecular Sciences, The University of New South Wales, Kensington, NSW, Australia; ^3^Marie Bashir Institute for Infectious Diseases and Biosecurity, The University of Sydney, Sydney, NSW, Australia; ^4^Centre for Infectious Diseases and Microbiology, Institute of Clinical Pathology and Medical Research, Westmead Hospital, Sydney Medical School, The University of Sydney, Westmead, NSW, Australia

**Keywords:** drug synergy, RNA-Seq, *Cryptococcus neoformans*, lactoferrin, amphotericin B, cellular stress response, *Saccharomyces*

## Abstract

Fungal infections are an increasing cause of morbidity and mortality. Current antifungal drugs are limited in spectrum, few new drugs are in development, and resistance is an increasing issue. Drug synergy can enhance available drugs and extend their lifetime, however, few synergistic combinations are in clinical use and mechanistic data on how combinations work is lacking. The multifunctional glycoprotein lactoferrin (LF) acts synergistically with amphotericin B (AMB) in a range of fungal species. Whole LF binds and sequesters iron, and LF can also be digested enzymatically to produce cationic peptides with distinct antimicrobial functions. To understand how LF synergizes AMB, we previously undertook a transcriptomic analysis in *Saccharomyces* and found a paradoxical down-regulation of iron and stress response, suggesting stress pathway interference was dysregulating an appropriate response, resulting in cell death. To extend this to a fungal pathogen, we here perform the same analysis in *Cryptococcus neoformans*. While both fungi responded to AMB in a similar way, the addition of LF produced remarkably contrasting results, with the *Cryptococcus* transcriptome enriched for processes relating to cellular stress, up-regulation of endoplasmic-reticulum-associated protein degradation (ERAD), stress granule disassembly and protein folding, endoplasmic reticulum-Golgi-vacuole trafficking and autophagy, suggesting an overall disruption of protein and lipid biosynthesis. These studies demonstrate that the mechanism of LF-mediated synergy is species-specific, possibly due to differences in the way LF peptides are generated, bind to and enter cells and act on intracellular targets, illustrating how very different cellular processes can underlie what appears to be a similar phenotypic response.

## Introduction

Pathogenic *Cryptococcus* species belonging to the *Cryptococcus neoformans* and *Cryptococcus gattii* species complexes can cause invasive disease in both healthy and immunocompromised people (Brown et al., 2012). Anti-cryptococcal therapy involves induction with amphotericin B (AMB) and 5-flucytosine (5-FC), followed by maintenance on fluconazole (FLC) (Perfect et al., 2010). However, mortality remains high, AMB and 5-FC are toxic, and 5-FC is not registered in many countries where the burden of cryptococcosis is highest (Brown et al., 2012; Day et al., 2013; Abassi et al., 2015).

Although it is widely recognized that there is an urgent need for new antifungal therapies, development is hindered by high risk and cost combined with relatively limited revenue (Santos-Gandelman and Machado-Silva, 2019). An alternative approach that aims to get more life out of existing drugs is drug synergy, where an antifungal is combined with a second drug or agent. The AMB-5FC combination used for cryptococcosis is a model example of improved antifungal efficacy, however, synergistic combinations are not widely employed in medical mycology (Day et al., 2013).

Previously, we tested a range of commonly used antifungal drugs with various iron chelating agents to determine whether iron limitation might enhance antifungal potency. Synergistic combinations were rare, however, the combination of AMB with lactoferrin (LF), a mammalian glycoprotein, produced a synergistic response in *Saccharomyces cerevisiae* and various *Cryptococcus* species, which had also been reported in *Candida* and *Aspergillus* (Venkatesh and Rong, 2008; Zarember et al., 2009). We further demonstrated that AMB-LF synergy was not due to iron starvation, as the addition of iron rescued the yeast cells from inhibition by LF but not from AMB-LF (Lai et al., 2016). To understand the mechanism of synergy, we performed comparative analyses of the transcriptomic responses to AMB and AMB-LF in *S. cerevisiae*. Paradoxically, we found that while AMB alone increased the expression of stress- and metal-related genes, the AMB-LF combination caused metal and stress-response transcripts to decrease. We hypothesized that AMB-LF synergy was mediated by dysregulation of metal homeostasis and disrupted stress responses, possibly via stress pathway interference (Pang et al., 2017).

To follow up this analysis in the context of a pathogenic yeast, we here report a companion transcriptomic analysis of AMB-LF synergy in *C. neoformans* strain H99. We show that, in contrast to *S. cerevisiae*, synergy is mediated by an enhanced stress response in *Cryptococcus* that includes disruption of endoplasmic reticulum-related processes. This study highlights the species-specific nature of responses to toxic agents by different fungal species, even when these appear to generate similar outcomes.

## Materials and Methods

### Strains, Culture and Agents

*Cryptococcus neoformans* strain H99 was cultured in yeast nitrogen broth (YNB) at 37°C with shaking at 180 rpm. Stock solutions of 1,600 μg/mL AMB (Sigma-Aldrich, United States) and 5,120 μg/mL LF (MP Biomedical, United States) were made in MilliQ water.

### Time Kill-Curves and RNA Extraction

Time-kill curves for AMB only, LF only and AMB-LF treatment were performed to determine ID_20_ (inhibition of cell growth by 20%) for RNA extraction and comparative transcriptomic analyses [as performed in Pang et al. (2017)]. Synergistic fractional inhibitory concentrations (FIC) of AMB-LF for *C. neoformans* [determined in Lai et al. (2016)] were used. ID_20_ was determined to be 1 h for AMB and 50 min for AMB-LF treatment. At these timepoints LF treatment did not affect growth (Supplementary Figure S1). RNA was extracted from the following cultures: (i) AMB treatment; (ii) growth control for (i); (iii) AMB-LF treatment; and (iv) growth control for (iii). Three independent biological replicates were performed for each treatment.

RNA was extracted from freeze dried cells using bead beating and the Qiagen RNeasy mini kit as detailed in Pang et al. (2017). RNA was sequenced at the Ramaciotti Centre for Genomics using an Illumina HiSeq 2000 to generate 100 bp paired-end reads.

### Processing of RNA-Seq Data

Data analysis was performed as in our previous study (Pang et al., 2017) but with updated software. Briefly, reads were mapped using the HISAT tool (Kim et al., 2015; version 2.1.0) to the *C. neoformans* var. *grubii* H99 reference genome (RefSeq accession: GCF_000149245.1). Reads counting was performed with the featureCount function of Subreads (Liao et al., 2014; R Core Team, 2019; version 3.5.3) with the edgeR library (R Core Team, 2019; version 3.24.3) and significant differentially expressed genes were defined as those with Benjamini and Hochberg (1995) adjusted *p*-value < 0.05. RUVSeq (Risso et al., 2014; version 1.16.1) was used to remove unwanted variation. After testing with zero to six unwanted factors with RUVSeq, the number of unwanted factors that maximized the number of significantly differentially expressed genes in total for each fungal species was used.

### Protein Orthologs and Gene Ontology Annotation

One-to-one protein orthologs from *S. cerevisiae* and *C. neoformans* were defined as reciprocal best hits from BLASTp searches (*E*-value < 1 × 10^–6^). The log fold-change of the unique ortholog pairs was graphed, a linear trend line was fitted, and Pearson’s correlation scores were calculated. Gene Ontology (GO) annotations were obtained from Uniprot (UniProt Consortium, 2019; release 2019_02), FungiDB (Basenko et al., 2018) and QuickGO (Binns et al., 2009; downloaded 10th April 2019). Additional GO annotations were obtained using Blast2GO (Gotz et al., 2008; version 5.25) and the Diamond sequence similar search tool (Buchfink et al., 2015; version 0.9.24) was used to compare the *C. neoformans* proteome against the Uniprot/Swiss-Prot database (UniProt Consortium, 2019; release 2019_02). Further annotations were obtained by transferring GO terms from orthologs of other fungal species using OrthoMCL (Fischer et al., 2011).

### Identifying Significantly Differentially Expressed Genes Unique to AMB or AMB-LF Treatments

Sets analysis was used to identify two mutually exclusive subsets of genes with significant differential expression unique to either AMB treatment or AMB-LF treatment. Each of these was then sub-divided to include genes with positive or negative log fold-change compared to the untreated control. The number of genes in each subset was represented as an UpSet plot (Lex et al., 2014). Each of the four subsets of genes was independently analyzed for enriched GO biological process terms using the methods described below.

### Gene Ontology Enrichment Analysis and Self-Organizing Maps

The “GOstats” R package and Fisher’s exact test (Falcon and Gentleman, 2007; version 2.48.0) were used for all GO enrichment analyses. Enriched GO terms were defined as those with Benjamini–Hochberg adjusted *p*-values < 0.05. For the global GO enrichment analysis, genes with increased or decreased expression were independently analyzed for enriched GO terms. Self-organizing map (SOM) analysis was used to identify clusters of significantly differentially expressed genes (adjusted *p*-values < 0.05) with similar expression profiles across the different treatments. The read counts in log counts per million were scaled to a mean of 0 and standard deviation of 1 across all samples per gene. Five-by-five SOM clusters were calculated using the R “kohonen” library (Wehrens and Kruisselbrink, 2018; version 3.0.8). Co-expressed genes from each SOM cluster were analyzed for enriched GO terms. To perform exploratory data analyses on the co-expression relationships and annotations of significantly differentially expressed genes (adjusted *p*-values < 0.05) in each SOM cluster, GO terms with moderate confidence of unadjusted *p*-values < 0.01 in each SOM cluster were analyzed in addition to the those that passed the stringent threshold of adjusted *p*-values < 0.05.

### Codes and Data Availability

*Cryptococcus neoformans* H99 RNA-Seq data generated from this study have been deposited in the NCBI Gene Expression Omnibus (GEO) (Edgar et al., 2002) and are accessible through GEO Series accession number GSE130375. *S. cerevisiae* S288C RNA-Seq data were previously described in Pang et al. (2017) and are accessible through GEO Series accession number GSE80357. The scripts for all the above analyses are available from Github: https://github.com/IgnatiusPang/Fungal_Drug_Synergy under the GNU Lesser General Public License v3.0.

## Results

### Differential Expression of *C. neoformans* Genes in Response to AMB and AMB-LF Treatment

We chose ID_20_ as the point to undertake transcriptome analysis, where cells should be responding to the primary effects of drug-related stress (Upadhya et al., 2013). *C. neoformans* cultures were treated with AMB and AMB-LF and harvested at their ID_20_, (60 min for AMB and 50 min for AMB-LF) along with their matched controls (Supplementary Figure S1). Initial QC analysis found one biological replicate of AMB-LF treatment was an outlier and it was excluded from subsequent analyses. In final analysis, the transcriptome of *Cryptococcus* following AMB treatment had 1625 up-regulated and 1957 down-regulated genes, relative to its matched control. Following AMB-LF treatment, 1599 genes were up-regulated while 1860 genes were down-regulated, compared to the matched control (Supplementary Table S1).

### AMB-LF Synergy Induces a Different Response in *C. neoformans* H99 and *S. cerevisiae* S288C

We previously performed a transcriptomic study of the response of *S. cerevisiae* to drug synergy induced by the addition of LF to AMB (Pang et al., 2017) (Supplementary Table S2). To compare this with the response by *C. neoformans*, genes that were differentially expressed following AMB and AMB-LF treatment (Supplementary Table S3) were analyzed for functional enrichment by GO (Pang et al., 2017) (Supplementary Tables S4, S5). In *C. neoformans*, the responses were similar across both treatments, with GO terms related to energy production, response to oxidative stress, carbohydrate metabolism, nucleic acid metabolism, protein processing and metal ion transport induced, and terms related to microtubule organization and lipid biosynthesis repressed. This contrasted with the situation in *S. cerevisiae*, where synergy between AMB and LF resulted in stress and metal homeostasis responses that were quite different to the effect of AMB treatment alone (Pang et al., 2017).

**FIGURE 1:**
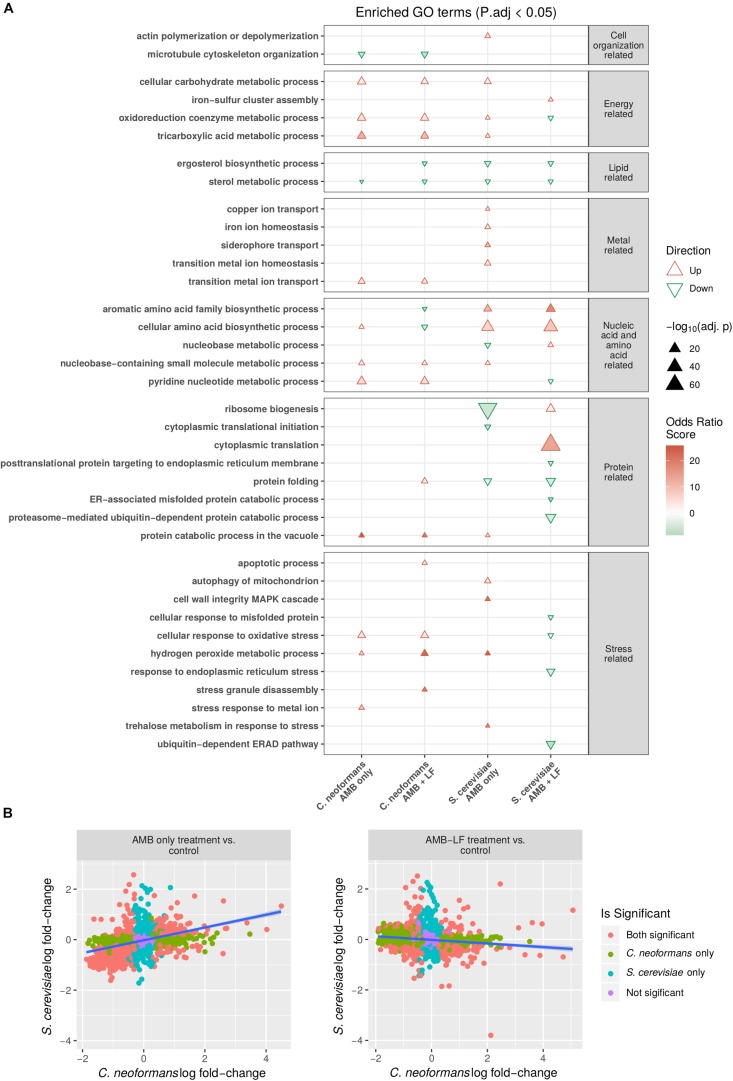
Transcriptomic and functional enrichment analyses highlight differences in the biological processes affected by AMB-LF synergy in *Cryptococcus neoformans* and *Saccharomyces cerevisiae*. **(A)** Enriched biological process GO terms based on differentially expressed genes in *C. neoformans* and *S. cerevisiae* in response to AMB and AMB-LF treatments. A subset of the enriched GO terms are listed on the *y*-axis, and similar GO terms are placed into groups including cell organization and replication-related, energy-related, lipid-related, metal-related, nucleic acid- and amino acid-related, protein related, and stress-related. The AMB and AMB-LF treatments in *C. neoformans* and *S. cerevisiae* are shown on the *x*-axis. Overall increased or decreased expression of each GO terms is indicated by a red triangle or green inverted triangle, respectively, with the size of the triangle scaled to represent the negative log adjusted *p*-value that shows the significance of the genes contributing to an enrichment relative to the total population of genes. Intensity of colors within the triangles indicate the magnitude of the odds ratio score, which represents the significance of the number of genes contributing to an enriched GO term. The two fungi responded to drug treatment quite differently, and in particular the synergy related to AMB-LF treatment induced opposite gene expressions for a number of processes, particularly protein folding and response to oxidative stress. **(B)** Comparison of the change in expression of genes mapped to protein sequence orthologs in *S. cerevisiae* and *C. neoformans*. Orthologs were defined as pairs of proteins with best hits from the reciprocal BLASTp searches of proteins from one organism to the proteome sequences of the other. The *y*-axis shows the log fold-change of *S. cerevisiae* ortholog transcripts, and the *x*-axis indicates the log fold-change of *C. neoformans* ortholog transcripts. Colored points indicate genes corresponding to protein orthologs that are significantly differentially expressed in both organisms (orange), *C. neoformans* only (green), *S. cerevisiae* only (blue), or not significant in either organism (purple). Linear trend is shown by a blue line. A significant positive correlation is seen for the AMB treatment (left) (ρ = 0.38, *p*-value < 0.05) while a negative correlation is seen for the AMB-LF treatment (ρ = –0.13, *p*-value < 0.05), suggesting synergy is mediated by different mechanisms in the two organisms.

Comparison of the response to AMB treatment found the majority of enriched biological processes that were common to both species had the same direction of regulation (Figure 1A). Enrichments related to energy generation, metal regulation and stress responses, amino acid biosynthesis and protein catabolism were all induced, while lipid metabolism was repressed. Functional enrichments that were absent from *C. neoformans* but were present in *S. cerevisiae* in response to AMB included the induction of autophagy, actin polymerization, iron homeostasis and copper transport, cell wall integrity MAPK cascade and trehalose metabolism in response to stress. Down-regulated enrichments that were observed only in *S. cerevisiae* included ergosterol biosynthesis, nucleobase metabolism, protein translation and folding-related processes.

In contrast to AMB, the transcriptional response to AMB-LF was drastically different in *C. neoformans* and *S. cerevisiae*. Functional enrichments related to energy generation processes, protein processing, response to stress and pyridine nucleotide metabolism were up-regulated in *C. neoformans* but down-regulated in *S. cerevisiae*, while amino acid metabolism was down-regulated in *C. neoformans* but up-regulated in *S. cerevisiae* (Figure 1A). Biological processes that were enriched in *C. neoformans* only included the up-regulation of apoptosis, transition metal transport, protein catabolism in the vacuole, stress granule assembly and hydrogen peroxide metabolism related to stress responses, and the down-regulation of microtubule organization. Enrichments that were absent in *C. neoformans* and specific to *S. cerevisiae* in response to AMB-LF were the induction of iron-sulfur cluster assembly, nucleobase metabolism and translation-related processes, and the repression of ER-associated processes including protein folding and ubiquitination, protein membrane localization, response to misfolded proteins and ER stress.

To verify that responses to AMB treatment were similar while responses to AMB-LF treatment differed between *C. neoformans* and *S. cerevisiae*, the log fold-change of genes expressed in *C. neoformans* and *S. cerevisiae* were plotted against one another and the correlation between genes that were orthologous between the two species were analyzed. The log fold-changes of orthologous genes following AMB treatment plotted in a positively correlated manner (ρ = 0.38, *p* < 0.05) suggesting similar responses (Figure 1B, left panel),while those in response to AMB-LF treatment were negatively correlated (ρ = −0.13, *p*-value < 0.05), demonstrating an overall opposing response by the two species to synergistic treatment (Figure 1B, right panel).

### Comparison of Gene Ontology Enrichments in *C. neoformans* Transcriptome Data Produced Following AMB and AMB-LF Treatment

While there were many similarities in the regulation of functional enrichments observed between AMB and AMB-LF treatments in *C. neoformans*, there were some GO enrichments that were unique to AMB-LF synergy (Figure 1A). These included down-regulation of ergosterol biosynthesis and aromatic amino acid family biosynthetic processes, and up-regulation of protein folding, stress granule disassembly and apoptotic processes. Cellular amino acid biosynthetic processes were up-regulated in response to AMB but down-regulated by AMB-LF. The functional enrichment that was seen only in AMB treatment was stress response to metal ion, and this was induced and involved genes encoding ABC transporters, a bile acid transporter, a zinc transporter and sulfide reductase (Supplementary Table S4).

**FIGURE 2:**
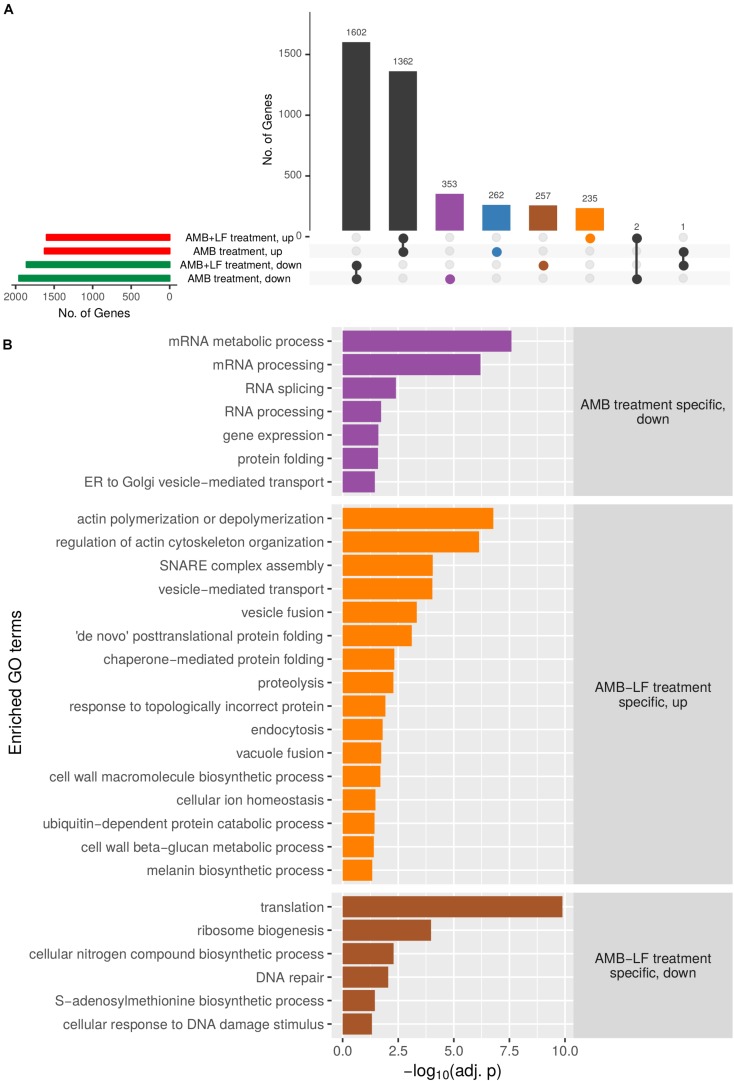
Upset plot analysis of genes differentially expressed in *C. neoformans* that are unique to AMB or AMB-LF treatment, and enriched biological process GO terms. **(A)** Sets analysis was used to analyze common and unique differentially expressed genes following AMB or AMB-LF treatments. The number of genes in each parameter (expressed in AMB/AMB-LF; up-/down-regulated, and their overlap) are represented as an UpSet plot (Lex et al., 2014). The horizontal bar graph on the left shows the number of genes with increased (red) or decreased (green) expression following AMB or AMB-LF treatments. The vertical bar graph shows the number of significantly differentially expressed genes (adjusted *p*-value < 0.05) from overlapping and non-overlapping parameters. Beneath the vertical bar graph is the combination matrix, where connected dots represent the overlap of parameters. The number of genes with significant changes in expression in both AMB and AMB-LF treatment are represented by black vertical bars. Differentially expressed genes unique to AMB treatment are represented by blue (up-regulated) and purple (down-regulated) bars, respectively, while unique AMB-LF genes are represented by orange (up-regulated) and brown (down-regulated) bars, respectively. **(B)** The four subsets of genes were independently analyzed to identify enriched biological process GO terms, with a representative listed on the *y*-axis. The *x*-axis indicates the level of enrichment, given as the negative log of the adjusted *p*-value. Genes with increased expression in AMB-LF treatment only (orange) highlight processes relating to the ER, Golgi and vacuole, including protein-folding, proteolysis, endocytosis, actin cytoskeleton organization, vacuole fusion, and cellular ion homeostasis. Decreased expression unique to AMB-LF treatment (brown) includes protein translation and DNA repair processes, indicating a slowing of DNA replication and translation. AMB treatment (purple) resulted in decreased expression of genes involved in transcription, translation and ER-to-Golgi transport. No biological enrichments were found for up-regulated genes unique to AMB treatment. The results suggest repression of ER-to-Golgi transport in response to AMB treatment, with an induction in ER and Golgi activity upon AMB-LF treatment.

To further tease out differences between AMB and AMB-LF, genes that were differentially expressed and present only in the response to AMB or to AMB-LF were separately analyzed for GO enrichments and are presented visually in an UpSets plot (Figure 2 and Supplementary Tables S3, S4). The sets analysis found a majority of differentially expressed genes to be shared between the AMB and AMB-LF treatment and identified transcripts that were unique to each (Figure 2A). Each group containing unique transcripts was analyzed to identify enriched biological process GO terms and their level of enrichment (Figure 2B). Functionally enriched GO terms that encompassed AMB-LF-specific genes mainly involved down-regulated processes related to protein synthesis (ribosome biogenesis and translation), amino acid biosynthesis (nitrogen compound and S-adenosyl methionine metabolism), and nucleic acid metabolism (RNA transport and response to DNA damage and repair). Up-regulated enrichments were mainly involved in actin polymerization and organization, membrane fusion (endocytosis, SNARE complex assembly, vesicle, and vacuolar fusion) and vesicle mediated transport, as well as ER and protein related processes (protein folding and proteolysis) and cell wall biosynthesis (glucan and melanin synthesis). Genes that were differentially regulated only in response to AMB had enrichments related to the down-regulation of RNA processing, protein folding and ER-to-Golgi transport (Figure 2). There were no biological enrichments identified among the AMB treatment up-regulated genes (Supplementary Table S4).

### SOMs Analysis Indicates AMB-LF Disrupts Iron Transmembrane Transport in *C. neoformans*

**FIGURE 3:**
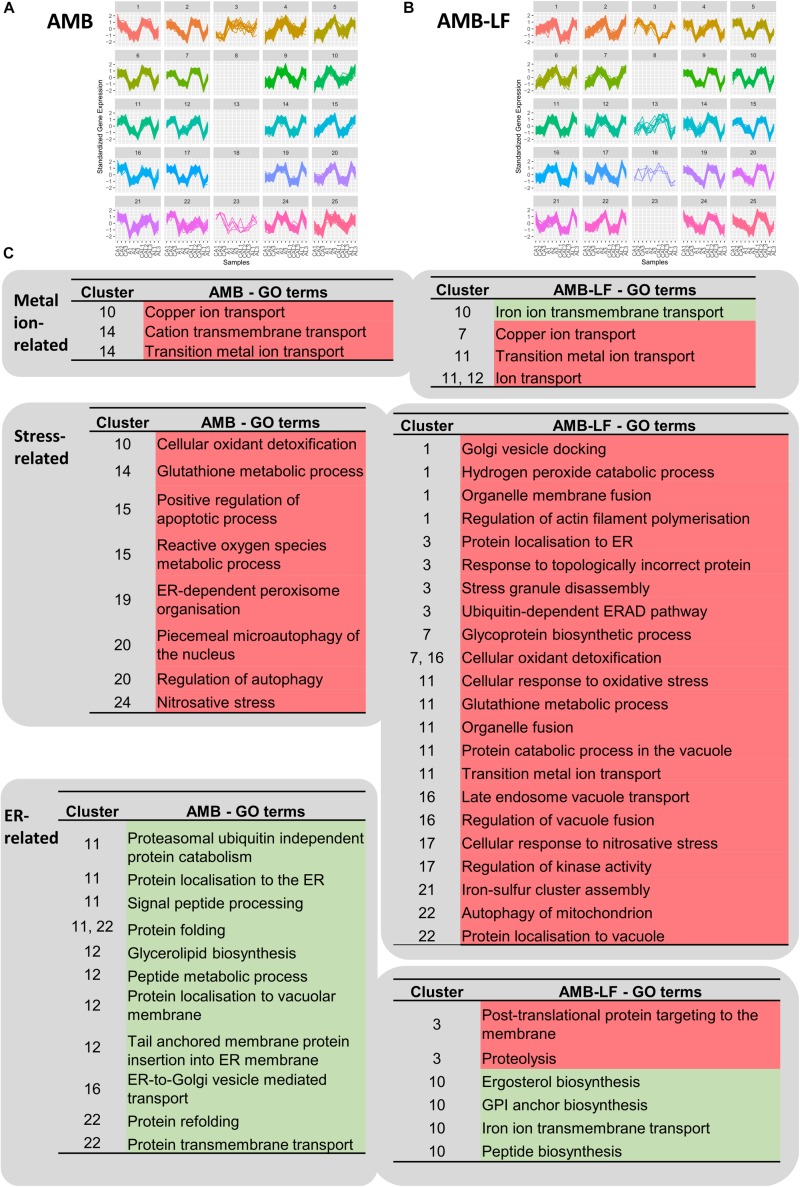
Self-organizing map (SOM) analysis of transcripts related to metal ions, stress and the endoplasmic reticulum. Independent SOMs are used to group co-expressed genes that were differentially expressed in *C. neoformans* in response to **(A)** AMB and **(B)** AMB-LF treatment. For each SOMs cluster, the *y*-axis denotes the standardized gene expression levels and the *x*-axis shows the 11 experimental samples: biological replicates of the control for AMB treatment (CA1-3), AMB treatment (A1-3), control for AMB-LF treatment (CAL1-3), and AMB-LF treatment (AL1-2). Each differentially expressed gene is represented by a line within each cluster, with clusters grouping genes with similar expression profiles that may be co-expressed and therefore potentially co-regulated. Clusters are organized and numbered from top left to bottom right. Empty clusters have no transcripts. **(C)** Enriched GO terms for AMB (left) and AMB-LF (right) treatment that are related to metal ions, stress and ER, and their associated SOMs clusters. Under AMB-LF synergy, stress responses occur across multiple clusters indicating a generalized cellular stress response, and these are linked with ER-Golgi-vacuole trafficking and proteolysis, suggesting a disruption of protein and lipid synthesis, sorting and trafficking. Further details of the GO terms and genes associated with clusters are given in Supplementary Tables S6, S7.

To investigate whether differentially expressed genes were co-expressed, we grouped transcripts with similar expression patterns using SOMs and analyzed these for the enrichment of biological process GO terms (Figure 3 and Supplementary Tables S6, S7). From this, we focused on clusters that included responses related to metal homeostasis, stress and ER-associated functions (Figure 3B), which we had found to differ between AMB and AMB-LF treatment (Figures 1, 2).

Self-organizing maps analysis indicated a general up-regulation of genes in enrichments related to metal homeostasis for both AMB and AMB-LF treatments. Enrichments related to copper and metal ion transport were seen for both treatments, with the genes making up the enrichments encoding iron, zinc and copper transporters (Figure 3B). There was down-regulation of genes related to iron ion transmembrane transport in AMB-LF that was not seen with AMB alone. These genes were co-expressed with protein and ergosterol synthesis in cluster 10 of the AMB-LF SOM (Figure 3B), suggesting a disruption in the synthesis of iron transport proteins.

### AMB-LF Synergy Increases Global Cellular Stress in *C. neoformans* and This Is Associated With Golgi and ER-Related Processes

For both AMB and AMB-LF treatment, analysis of co-expressed enrichments found stress-related responses to be induced and enriched across multiple different SOMs clusters. For example, in the AMB response terms related to free radical detoxification, oxidative and nitrosative stress and glutathione metabolism were enriched in clusters 10, 14, 15, 19, and 24, and similar terms were enriched in clusters 1, 7, 11, 16, and 17 in the AMB-LF response (Figure 3B). This suggests that stress is generalized across the cell and is not associated with any particular biological process.

Similar to previous studies, our analysis indicated that AMB induces responses linked to oxidative and nitrosative stress in *C. neoformans* (Ferreira et al., 2013). ER-dependent peroxisome organization, where peroxisomes form in response to hydrogen peroxide production from fatty oxidation, was also induced in response to AMB (cluster 19) along with autophagy (cluster 20) and apoptotic processes (cluster 15).

Oxidative and nitrosative stress, glutathione metabolism and autophagy were likewise induced in response to AMB-LF (clusters 1, 7, 11, 16, 17, and 22), however, these and other stress responses were co-enriched with processes related to protein synthesis and transit through the ER (Figure 3B). Induction of the enrichment related to stress granules (cluster 3), which are assemblies of untranslated messenger ribonucleoproteins (Protter and Parker, 2016), suggests stress may be resulting from disrupted protein translation. ER stress was further suggested in cluster 3 by induced enrichments related to topologically incorrect proteins and the ERAD (ER-associated protein degradation) response, which is activated by the accumulation of misfolded proteins (Stolz and Wolf, 2010). Across other SOMs clusters, enrichments related to Golgi-related protein processing and trafficking to the vacuole, such as Golgi vesicle docking (cluster 1), glycoprotein biosynthesis (cluster 7), endosome vacuole transport (cluster 16), and protein catabolism in the vacuole (cluster 11) were induced and associated with stress responses. The overall relation of stress responses across different clusters and biological processes indicates that AMB-LF treatment induces substantially more stress than AMB alone. Although enrichments related to the induction of apoptosis were not evident in the AMB-LF SOMs data, apoptosis was up-regulated in the functional enrichment profile for this treatment overall (Figure 1), further supporting the notion that the addition of LF to AMB substantially enhances cellular stress.

### Addition of LF to AMB Disrupts Biosynthetic, Folding, Sorting and Transport Processes Undertaken by the ER and Golgi and Induces Protein Trafficking

ER- and Golgi-related processes were enriched across different SOMs clusters following both AMB and AMB-LF treatment (Figure 3). For AMB treatment, these enrichments likely reflect a down-regulation in protein sorting and trafficking through the secretory pathway, and include localization of protein to the ER (cluster 11), ER processes such as glycerolipid biosynthesis, protein folding and signal peptide processing for translocation (cluster 11, 12, and 22), and vesicle mediated transport to the Golgi (cluster 16). This AMB-mediated repression of protein biosynthesis and sorting processes in *C. neoformans* is consistent with the transcriptome responses to AMB seen for *S. cerevisiae* and *C. albicans* (Zhang et al., 2002; Agarwal et al., 2003; Liu et al., 2005). Other enrichments associated with proteasomal protein degradation and localization to the vacuole membrane were also down-regulated in clusters 11 and 12.

Up- and down-regulation of ER- and Golgi-related functional enrichments were observed in response to AMB-LF treatment (Figure 3B). Similar to AMB treatment, ER- and Golgi-related processes were repressed, including synthesis of lipids, peptides, glycosylphosphatidylinositol (GPI) anchors and proteins involved in iron transport that require GPI modifications, which were all co-expressed in cluster 10. The exception was glycoprotein synthesis, which was induced in cluster 7. Unlike the response to AMB treatment, however, protein trafficking was induced and SOMs enrichments reflected three routes of transport from the ER and Golgi that were co-enriched with stress responses. ER-to-Golgi transport through the secretory pathway was suggested by enrichments involving localization of proteins to the ER and Golgi vesicle docking in clusters 1 and 3. The enrichment relating to endosome-to-vacuole transport in cluster 16 suggests Golgi-to-vacuole trafficking of proteins for storage or degradation. ER-to-cytosol transport-related enrichments were co-enriched in cluster 3 and reflect ERAD activation, which catalyzes the transport of misfolded proteins between the ER and the cytosol for proteolysis (Stolz and Wolf, 2010). The difference in regulation of ER and Golgi functions seen between AMB and AMB-LF treatments suggests that the addition of LF to AMB disrupts cellular protein and lipid synthesis, with misfolded proteins inducing sorting and trafficking.

## Discussion

**FIGURE 4:**
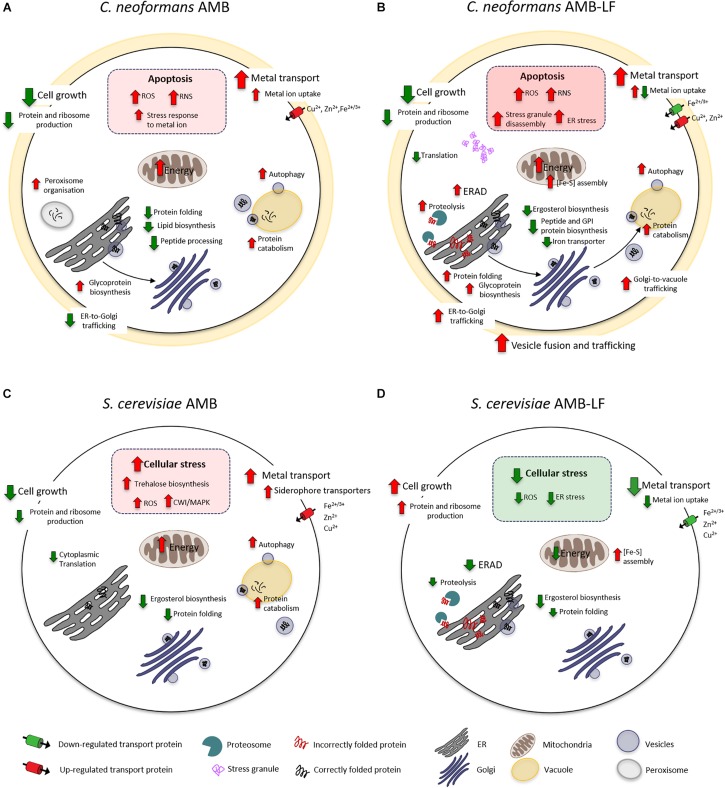
A model of AMB-LF synergy in *Cryptococcus neoformans* and *Saccharomyces cerevisiae*. Diagrams of the cellular response to AMB treatment and AMB-LF treatment in *C. neoformans* **(A,B)** and *S. cerevisiae* **(C,D)**. In all cases the transcriptome was analyzed at ID_20_ relative to the untreated control. While the response to AMB was similar overall in both species **(A,C)**, AMB-LF synergy was mediated by opposing processes. The up-regulation of stress-related processes in *C. neoformans* suggests cells are killed by overwhelming cellular stress **(B)**, while down-regulation of these processes and up-regulation of cell growth in *S. cerevisiae* suggests the addition of LF prevents the cell from mounting an appropriate stress response **(D)**.

The current study follows our analysis of AMB-LF synergy in *S. cerevisiae*, which was undertaken to allow a detailed analysis of synergy using the extensive genetic and bioinformatic resources that are available for yeast. We chose ID_20_ for both studies, where growth had been inhibited by 20% compared to the untreated control, and hypothesized that synergy would be mediated by similar mechanisms that could be used to find novel drug targets that are conserved across fungal species (Pang et al., 2017). However, to our surprise we have found synergy to occur via two different and opposing mechanisms, with dysregulation of the metal ion response and down-regulation of stress in *Saccharomyces*, and up-regulation of cellular stress in *Cryptococcus*. For the former, we suggested that the independent assaults by AMB and LF resulted in stress pathway interference, preventing the *S. cerevisiae* cells from sensing and responding to toxic stress in an appropriate way (Pang et al., 2017). For *Cryptococcus*, we propose that the combined agents result in excessive oxidative and nitrosative damage and an up-regulation of autophagy that results from increased ERAD and ER-Golgi-vacuole trafficking and disrupted protein and lipid biosynthesis. A model of the responses of both species to AMB and AMB-LF that summarizes the data overall and the results shown in Figures 1–3 is presented in Figure 4.

Amphotericin B has a broad spectrum of activity against yeasts and molds. AMB binds membrane ergosterol, and it is widely accepted that antifungal activity is mediated by the disruption of cellular integrity (Mesa-Arango et al., 2012). However, recent studies in yeast indicate that AMB can also enter cells and uses autophagy-dependent transport to the vacuole, which it then damages and disrupts (Yoshioka et al., 2016). AMB also induces oxidative and nitrosative stress (Liu et al., 2005; Ferreira et al., 2013), and killing in *C. neoformans* involves induction of an oxidative burst (Sangalli-Leite et al., 2011). Our transcriptomic data for AMB alone are in good agreement with these studies, with induction of ROS and the overall stress response and an up-regulation of autophagy and vacuole-mediated protein catabolism seen in both *Cryptococcus* and *Saccharomyces*. In addition, AMB caused an up-regulation of genes related to metal ion transport and energy production and down-regulation of cell growth, protein and ribosome production and ergosterol biosynthesis in both species (Figures 4A,C), which are strong indicators of cellular inhibition and response to membrane stress (Liu et al., 2005).

Like AMB, LF appears to bind to and disrupt cell membranes, which together with the ability to sequester iron is important in its antimicrobial activity. Unbiased ‘omics approaches have not been used to study the fungal response to LF, and at the FIC levels used here LF does not affect the transcriptome (Pang et al., 2017). However, analysis of the apoptotic response of *Saccharomyces* to LF found inhibition requires *de novo* protein synthesis and energy, with LF causing mitochondrial dysfunction resulting in ROS accumulation (Acosta-Zaldívar et al., 2016). LF targeting of ER and Golgi processes has not been assessed in current literature. However, our finding of up-regulated terms related to ERAD and vesicle trafficking with autophagy, which were co-expressed and linked to the up-regulation of misfolded proteins and proteolysis and the accumulation of stress granules, together with the down-regulation of ergosterol, GPI anchor and iron transporter synthesis (Figure 4B), suggest disrupted protein and lipid biosynthesis are likely to be an important component of LF-mediated synergy in *Cryptococcus*.

Also in common with AMB, LF is a natural product with multiple biological functions (Fernandes and Carter, 2017). In addition to being antimicrobial, LF has anti-tumor and anti-oxidant properties and it can both stimulate and repress the inflammatory response to infection (Drago-Serrano et al., 2017), presumably by differential binding to receptors on target cell surfaces and subsequent uptake (Suzuki et al., 2005). There is emerging evidence that following binding, LF can enter microbial cells and interact directly with intracellular targets (Frontera et al., 2018). Furthermore, proteolysis results in the production of cationic peptides, in particular lactoferricin and lactoferrampin, that have even greater antimicrobial activity than intact LF (Sinha et al., 2013). Thus, LF activity toward fungi is likely to be complex and multifactorial: secreted fungal enzymes may digest LF to a greater or lesser extent, and whole LF along with lactoferricin, lactoferrampin and potentially other peptides may interact with variable receptors on the fungal cell membrane and disrupt a range of intracellular processes. Any of these processes may differ between *Saccharomyces* and *Cryptococcus* and account for their opposing responses to AMB-LF synergy.

The ‘omics approach used here provides a whole-of-organism view of the response to the drug combination (Mack et al., 2018), and clustering transcripts by SOMs identified co-expressed genes that enable identification of stress-response pathways (Hudson et al., 2012). However, the transcriptome is an indirect assessment of cellular changes, and validation studies are required to determine if the assignment of biological terms based on differential gene expression translates to actual cellular processes. With the pressing need to find new ways to treat fungal infections, understanding how synergy works will be valuable for both augmenting existing therapies and for developing novel, effective antifungal strategies.

## Conclusion

We conclude that a phenotypically similar outcome to drug synergy is mediated by distinctly different pathways in *Saccharomyces* and *Cryptococcus*. The current study indicates that the combined assaults of AMB and LF produce overwhelming stress to the *C. neoformans* cell due to a disruption of protein and lipid biosynthesis that results in the up-regulation of ERAD, trafficking from the ER to the Golgi to the vacuole, with autophagy and apoptosis. The current study suggests LF or LF-derived peptides have potential to augment AMB and lower the required dose, thereby both improving efficacy and reducing toxicity.

## Data Availability Statement

The datasets generated for this study can be found in the NCBI Gene Expression Omnibus (GEO) accession number GSE130375.

## Author Contributions

DC, MW, and SC conceived the study, obtained grant funding, oversaw the work, and assisted with the data analysis. Y-WL undertook all laboratory work and analysis with supervision from DC, LC, and SC. CP undertook all bioinformatic analyses with supervision from MW. Y-WL, CP, and DC drafted the manuscript. MW, SC, and LC commented and edited the drafts. All authors read and approved the submitted version of the manuscript.

## Conflict of Interest

The authors declare that the research was conducted in the absence of any commercial or financial relationships that could be construed as a potential conflict of interest.

## References

[B1] Abassi M. Boulware D. R. Rhein J. (2015). Cryptococcal meningitis: diagnosis and management update. *Curr. Trop. Med. Rep.* 2 90–99. 10.1007/s40475-015-0046-y 26279970 PMC4535722

[B2] Acosta-Zaldívar M. Andrés M. T. Rego A. Pereira C. S. Fierro J. F. Côrte-Real M. (2016). Human lactoferrin triggers a mitochondrial- and caspase-dependent regulated cell death in *Saccharomyces cerevisiae*. *Apoptosis* 21 163–173. 10.1007/s10495-015-1199-9 26577769

[B3] Agarwal A. K. Rogers P. D. Baerson S. R. Jacob M. R. Barker K. S. Cleary J. D. (2003). Genome-wide expression profiling of the response to polyene, pyrimidine, azole, and echinocandin antifungal agents in *Saccharomyces cerevisiae*. *J. Biol. Chem.* 278 34998–35015. 10.1074/jbc.m306291200 12824174

[B4] Basenko E. Y. Pulman J. A. Shanmugasundram A. Harb O. S. Crouch K. Starns D. (2018). FungiDB: an integrated bioinformatic resource for fungi and oomycetes. *J. Fungi* 4:E39. 10.3390/jof4010039 30152809 PMC5872342

[B5] Benjamini Y. Hochberg Y. (1995). Controlling the false discovery rate: a practical and powerful approach to multiple testing. *J. R. Stat. Soc. Series B Methodol.* 57 289–300. 10.1111/j.2517-6161.1995.tb02031.x

[B6] Binns D. Dimmer E. Huntley R. Barrell D. O’Donovan C. Apweiler R. (2009). QuickGO: a web-based tool for gene ontology searching. *Bioinformatics* 25 3045–3046. 10.1093/bioinformatics/btp536 19744993 PMC2773257

[B7] Brown G. D. Denning D. W. Gow N. A. R. Levitz S. M. Netea M. G. White T. C. (2012). Hidden killers: human fungal infections. *Sci. Transl. Med.* 4:165rv13. 10.1126/scitranslmed.3004404 23253612

[B8] Buchfink B. Xie C. Huson D. H. (2015). Fast and sensitive protein alignment using diamond. *Nat. Methods* 12 59–60. 10.1038/nmeth.3176 25402007

[B9] Day J. N. Chau T. T. H. Wolbers M. Mai P. P. Dung N. T. Mai N. H. (2013). Combination antifungal therapy for cryptococcal meningitis. *N. Engl. J. Med.* 368 1291–1302. 23550668 10.1056/NEJMoa1110404 PMC3978204

[B10] Drago-Serrano M. Campos-Rodríguez R. Carrero J. De La Garza M. (2017). Lactoferrin: balancing ups and downs of inflammation due to microbial infections. *Int. J. Mol. Sci.* 18:E501. 10.3390/ijms18030501 28257033 PMC5372517

[B11] Edgar R. Domrachev M. Lash A. E. (2002). Gene expression omnibus: NCBI gene expression and hybridization array data repository. *Nucleic Acids Res.* 30 207–210. 10.1093/nar/30.1.207 11752295 PMC99122

[B12] Falcon S. Gentleman R. (2007). Using GOstats to test gene lists for GO term association. *Bioinformatics* 23 257–258. 10.1093/bioinformatics/btl567 17098774

[B13] Fernandes K. E. Carter D. A. (2017). The antifungal activity of lactoferrin and its derived peptides: mechanisms of action and synergy with drugs against fungal pathogens. *Front. Microbiol.* 8:2. 10.3389/fmicb.2017.00002 28149293 PMC5241296

[B14] Ferreira G. F. Baltazar Lde M. Santos J. R. Monteiro A. S. Fraga L. A. Resende-Stoianoff M. A. (2013). The role of oxidative and nitrosative bursts caused by azoles and amphotericin B against the fungal pathogen *Cryptococcus gattii*. *J. Antimicrob. Chemother.* 68 1801–1811. 10.1093/jac/dkt114 23612570

[B15] Fischer S. Brunk B. P. Chen F. Gao X. Harb O. S. Iodice J. B. (2011). Using OrthoMCL to assign proteins to OrthoMCL-DB groups or to cluster proteomes into new ortholog groups. *Curr. Protoc. Bioinformatics* 12 1–19. 10.1002/0471250953.bi0612s35 PMC3196566 21901743

[B16] Frontera L. S. Moyano S. Quassollo G. Lanfredi-Rangel A. Rópolo A. S. Touz M. C. (2018). Lactoferrin and lactoferricin endocytosis halt *Giardia* cell growth and prevent infective cyst production. *Sci. Rep.* 8:18020. 10.1038/s41598-018-36563-1 30575774 PMC6303297

[B17] Gotz S. Garcia-Gomezm J. M. Terol J. Williams T. D. Nagaraj S. H. Nueda M. J. (2008). High-throughput functional annotation and data mining with the Blast2GO suite. *Nucleic Acids Res.* 36 3420–3435. 10.1093/nar/gkn176 18445632 PMC2425479

[B18] Hudson N. J. Dalrymple B. P. Reverter A. (2012). Beyond differential expression: the quest for causal mutations and effector molecules. *BMC Genomics* 13:356. 10.1186/1471-2164-13-356 22849396 PMC3444927

[B19] Kim D. Langmead B. Salzberg S. L. (2015). HISAT: a fast spliced aligner with low memory requirements. *Nat. Methods* 12 357–360. 10.1038/nmeth.3317 25751142 PMC4655817

[B20] Lai Y. W. Campbell L. T. Wilkins M. R. Pang C. N. Chen S. Carter D. A. (2016). Synergy and antagonism between iron chelators and antifungal drugs in *Cryptococcus*. *Int. J. Antimicrob. Agents* 48 388–394. 10.1016/j.ijantimicag.2016.06.012 27474467

[B21] Lex A. Gehlenborg N. Strobelt H. Vuillemot R. Pfister H. (2014). UpSet: visualization of intersecting sets. *IEEE Trans. Vis. Comput. Graph.* 20 1983–1992. 10.1109/TVCG.2014.2346248 26356912 PMC4720993

[B22] Liao Y. Smyth G. K. Shi W. (2014). Featurecounts: an efficient general purpose program for assigning sequence reads to genomic features. *Bioinformatics* 30 923–930. 10.1093/bioinformatics/btt656 24227677

[B23] Liu T. T. Lee R. E. Barker K. S. Lee R. E. Wei L. Homayouni R. (2005). Genome-wide expression profiling of the response to azole, polyene, echinocandin, and pyrimidine antifungal agents in *Candida albicans*. *Antimicrob. Agents Chemother.* 49 2226–2236. 10.1128/aac.49.6.2226-2236.2005 15917516 PMC1140538

[B24] Mack S. G. Turner R. L. Dwyer D. J. (2018). Achieving a predictive understanding of antimicrobial stress physiology through systems biology. *Trends Microbiol.* 26 296–312. 10.1016/j.tim.2018.02.004 29530606

[B25] Mesa-Arango A. C. Scorzoni L. Zaragoza O. (2012). It only takes one to do many jobs: amphotericin B as antifungal and immunomodulatory drug. *Front. Microbiol.* 3:286. 10.3389/fmicb.2012.00286 23024638 PMC3441194

[B26] Pang C. N. Lai Y. W. Campbell L. T. Chen S. C. Carter D. A. Wilkins M. R. (2017). Transcriptome and network analyses in *Saccharomyces cerevisiae* reveal that amphotericin B and lactoferrin synergy disrupt metal homeostasis and stress response. *Sci. Rep.* 7:40232. 10.1038/srep40232 28079179 PMC5228129

[B27] Perfect J. R. Dismukes W. E. Dromer F. Goldman D. L. Graybill J. R. Hamill R. J. (2010). Clinical practice guidelines for the management of cryptococcal disease: 2010 update by the Infectious diseases society of America. *Clin. Infect. Dis.* 50 291–322. 10.1086/649858 20047480 PMC5826644

[B28] Protter D. S. W. Parker R. (2016). Principles and properties of stress granules. *Trends Cell Biol.* 26 668–679. 10.1016/j.tcb.2016.05.004 27289443 PMC4993645

[B29] R Core Team (2019). *R: A Language and Environment for Statistical Computing.* Vienna: R Foundation for Statistical Computing.

[B30] Risso D. Ngai J. Speed T. P. Dudoit S. (2014). Normalization of RNA-seq data using factor analysis of control genes or samples. *Nat. Biotechnol.* 32 896–902. 10.1038/nbt.2931 25150836 PMC4404308

[B31] Sangalli-Leite F. Scorzoni L. Mesa-Arango A. C. Casas C. Herrero E. Gianinni M. J. (2011). Amphotericin B mediates killing in *Cryptococcus neoformans* through the induction of a strong oxidative burst. *Microbes Infect.* 13 457–467. 10.1016/j.micinf.2011.01.015 21310262

[B32] Santos-Gandelman J. Machado-Silva A. (2019). Drug development for cryptococcosis treatment: what can patents tell us? *Mem. Inst. Oswaldo Cruz* 114:e180391. 10.1590/0074-02760180391 30726342 PMC6358010

[B33] Sinha M. Kaushik S. Kaur P. Sharma S. Singh T. P. (2013). Antimicrobial lactoferrin peptides: the hidden players in the protective function of amultifunctional protein. *Int. J. Pept.* 2013 1–12. 10.1155/2013/390230 23554820 PMC3608178

[B34] Stolz A. Wolf D. H. (2010). Endoplasmic reticulum associated protein degradation: a chaperone assisted journey to hell. *Biochim. Biophys. Acta Mol. Cell Res.* 1803 694–705. 10.1016/j.bbamcr.2010.02.005 20219571

[B35] Suzuki Y. A. Lopez V. Lönnerdal B. (2005). Mammalian lactoferrin receptors: structure and function. *Cell. Mol. Life Sci.* 62 2560–2575. 10.1007/s00018-005-5371-1 16261254 PMC11139119

[B36] UniProt Consortium (2019). UniProt: a worldwide hub of protein knowledge. *Nucleic Acids Res.* 47 D506–D515. 10.1093/nar/gky1049 30395287 PMC6323992

[B37] Upadhya R. Campbell L. T. Donlin M. J. Aurora R. Lodge J. K. (2013). Global transcriptome profile of *Cryptococcus neoformans* during exposure to hydrogen peroxide induced oxidative stress. *PLoS One* 8:e55110. 10.1371/journal.pone.0055110 23383070 PMC3557267

[B38] Venkatesh M. P. Rong L. (2008). Human recombinant lactoferrin acts synergistically with antimicrobials commonly used in neonatal practice against coagulase-negative staphylococci and *Candida albicans* causing neonatal sepsis. *J. Med. Microbiol.* 57 1113–1121. 10.1099/jmm.0.2008/001263-0 18719181

[B39] Wehrens R. Kruisselbrink J. (2018). Flexible Self-Organizing Maps in kohonen 3.0. *J. Stat. Softw.* 87 1–18.

[B40] Yoshioka M. Yamada K. Yamaguchi Y. Ogita A. Fujita K.-I. Tanaka T. (2016). The fungicidal activity of amphotericin B requires autophagy-dependent targeting to the vacuole under a nutrient-starved condition in *Saccharomyces cerevisiae*. *Microbiology* 162 848–854. 10.1099/mic.0.000269 26940206

[B41] Zarember K. A. Cruz A. R. Huang C.-Y. Gallin J. I. (2009). Antifungal activities of natural and synthetic iron chelators alone and in combination with azole and polyene antibiotics against *Aspergillus fumigatus*. *Antimicrob. Agents Chemother.* 53 2654–2656. 10.1128/AAC.01547-08 19307370 PMC2687209

[B42] Zhang L. Zhang Y. Zhou Y. An S. Zhou Y. Cheng J. (2002). Response of gene expression in *Saccharomyces cerevisiae* to amphotericin B and nystatin measured by microarrays. *J. Antimicrob. Chemother.* 49 905–915. 10.1093/jac/dkf001 12039882

