# Supplementary material for: Different Pathways Mediate Amphotericin-Lactoferrin Drug Synergy in Cryptococcus and Saccharomyces
Source: Front Microbiol. 2019 Oct 1;10:2195. doi: 10.3389/fmicb.2019.02195 (PMC6779777; doi:10.3389/fmicb.2019.02195)
Supplement: Supplementary file 1 [file Image_1.pdf]

**Supplementary Figure 1**

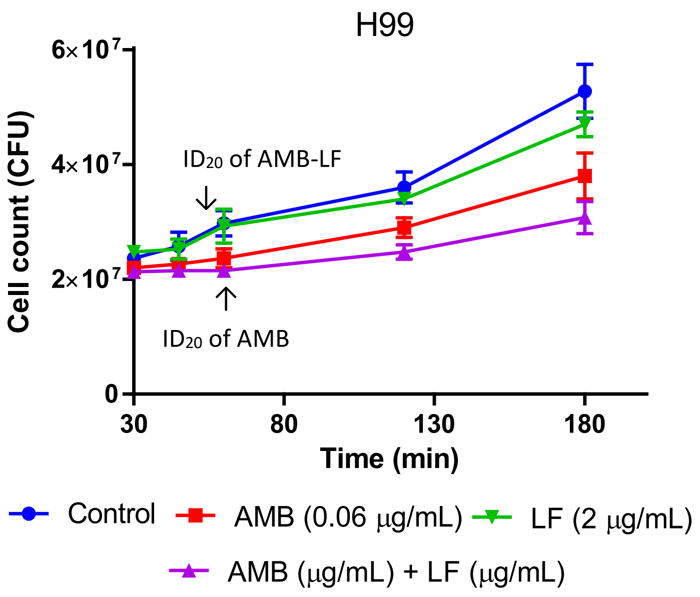


**Supplementary Figure 1. Inhibition of *C. neoformans* by AMB, LF and AMB-LF.** Drugs were added to a 50 mL culture containing 2 x $10^7$ cells/mL and samples were taken at 30, 45, 60, 120 and 180 min after the addition of drugs to assess level of inhibition. $ID_{20}$ occurred at 50 min for AMB-LF and 1 hr for AMB treatment. Data is shown as mean +/- standard error of the mean (SEM).
